# Using a generative adversarial network to generate synthetic MRI images for multi-class automatic segmentation of brain tumors

**DOI:** 10.3389/fradi.2023.1336902

**Published:** 2024-01-18

**Authors:** P. Raut, G. Baldini, M. Schöneck, L. Caldeira

**Affiliations:** ^1^ Department of Pediatric Pulmonology, Erasmus Medical Center, Rotterdam, Netherlands; ^2^ Department of Radiology & Nuclear Medicine, Erasmus Medical Center, Rotterdam, Netherlands; ^3^ Institute for Diagnostic and Interventional Radiology, University Hospital Cologne, Cologne, Germany; ^4^ Institute of Interventional and Diagnostic Radiology and Neuroradiology, University Hospital Essen, Essen, Germany

**Keywords:** deep learning, 3D convolutional neural network, generative adversarial network, synthetic images, multi-parametric MRI, brain tumors, segmentation

## Abstract

Challenging tasks such as lesion segmentation, classification, and analysis for the assessment of disease progression can be automatically achieved using deep learning (DL)-based algorithms. DL techniques such as 3D convolutional neural networks are trained using heterogeneous volumetric imaging data such as MRI, CT, and PET, among others. However, DL-based methods are usually only applicable in the presence of the desired number of inputs. In the absence of one of the required inputs, the method cannot be used. By implementing a generative adversarial network (GAN), we aim to apply multi-label automatic segmentation of brain tumors to synthetic images when not all inputs are present. The implemented GAN is based on the Pix2Pix architecture and has been extended to a 3D framework named Pix2PixNIfTI. For this study, 1,251 patients of the BraTS2021 dataset comprising sequences such as T_1_w, T_2_w, T_1_CE, and FLAIR images equipped with respective multi-label segmentation were used. This dataset was used for training the Pix2PixNIfTI model for generating synthetic MRI images of all the image contrasts. The segmentation model, namely DeepMedic, was trained in a five-fold cross-validation manner for brain tumor segmentation and tested using the original inputs as the gold standard. The inference of trained segmentation models was later applied to synthetic images replacing missing input, in combination with other original images to identify the efficacy of generated images in achieving multi-class segmentation. For the multi-class segmentation using synthetic data or lesser inputs, the dice scores were observed to be significantly reduced but remained similar in range for the whole tumor when compared with evaluated original image segmentation (e.g. mean dice of synthetic T_2_w prediction NC, 0.74 ± 0.30; ED, 0.81 ± 0.15; CET, 0.84 ± 0.21; WT, 0.90 ± 0.08). A standard paired *t*-tests with multiple comparison correction were performed to assess the difference between all regions (*p* < 0.05). The study concludes that the use of Pix2PixNIfTI allows us to segment brain tumors when one input image is missing.

## 1 Introduction

In a standard care protocol for glioblastoma, imaging is considered a crucial tool for the diagnosis and the monitoring of the patients. Magnetic resonance imaging (MRI) is one of the most widely chosen modalities among others by treating clinicians for tracing the progression of the disease. Several dedicated multi-parametric image acquisition sequences are used at the different stages of the disease, but T_1_-weighted (T_1_w), T_2_-weighted (T_2_w), contrast-enhanced T_1_w (T_1_CE), and FLAIR (fluid-attenuated inversion recovery) are one of the few types of imaging sequences commonly being used in a routine protocol (1, 2). These multi-parametric MRI (mpMRI) sequences are efficient in highlighting the biological status and smallest changes occurring in the tumor micro-environment, thereby guiding clinicians in providing the best care for the patient (3). To precisely locate and trace the progression of anomalies, to study the tumor micro-environment for strategic planning of treatment, and to determine the efficacy of applied treatment, the quantification of lesions is considered an essential step in a clinical establishment. However, manual volumetric segmentation of brain lesions is a challenging, repetitive, and time-consuming task. At the same time, it is dependent on the skills of the expert and knowledge of the subject and thus prone to intra-reader or inter-reader bias. Hence, the manual segmentation method is less reliable and precise with limited reproducibility and repeatability. The drawbacks of the manual method and the unmet need for having accurate and user-independent segmentation tools, which possess the potential to become an integral part of the clinical workflow, can be overcome by implementing automated algorithms for multi-regional segmentation.

Deep learning (DL), an advanced artificial intelligence (AI)-based technique, provides a solution for automatic analysis using three-dimensional convolutional neural networks (3D CNN) (4–6). These neural networks proved to have the potential to provide an automatic analysis based on training the algorithms with a variety of large-quantity datasets for achieving consistent and improvised results (7–9). The key to gaining highly precise results using 3D CNN is to train the algorithms with a large cohort of heterogeneous datasets. These CNNs allow the creation of application-based models for various purposes using different types of modalities, producing close accurate results which often outperform the manual analysis (10, 11). However, due to a great variation in the local pathologies of brain tumors, the algorithm must face several challenges to be clinically relevant, including being subjected to numerous anatomical disparities, with images acquired using different types of acquisition protocols across multiple scanners.

While employing CNN for automatic analysis, several strict prerequisites are expected to be met for the trained model to function properly. However, these prerequisites are not always matched in clinical practice, such as the number of input images required for the algorithm, which observed to be incomplete in most of the cases. Depending on the clinical indication, the image sequences are selected individually and can differ in number at every instance; hence, not all the required images for automatic segmentation are always available. This is one of the most common existing issues in clinical environments, which hinders the use of AI models in clinical practice. However, the availability of required multiple input images (T_1_w, T_2_w, T_1_CE, and FLAIR for the brain tumor segmentation) is crucial for the generation of predictions as it provides greater accuracy and precision for the detection of heterogeneous tissue structure within tumor micro-environment to draw multi-class segmentation. For example, T_1_w image aids in the detection of adipose tissue and tumor boundaries, while T_2_w–FLAIR accentuates vasculature within the tumor and T_1_CE highlights the tissue perfusion characteristics.

The challenge of missing input data can be logically addressed by generating the images using another DL-based technique making image-to-image translation (single-input/multi-input and single-output) on principles of generative adversarial networks (GANs). The method is widely used for creating an approximate image of the desired modality using another available image (12–14). However, it is yet unknown whether it is beneficial to use synthetic data in combination with other original images for deriving automatic segmentation using 3D CNN. To be clinically relevant, synthetic images should provide a sufficient approximation of anatomical variation of missing contrast to enable automatic analysis. Furthermore, the model to be deployed first must prove its ability to be reliable, robust, consistent, and time-efficient.

All of the deficiencies of existing clinical routines forbidding the use of AI models for automatic segmentation were taken into consideration in this study. The primary objective of this study was to develop a model based on existing CNN for accurate multi-regional automatic segmentation of brain tumors using a variable number of input channels for the training of the algorithm. The secondary objective involved the evaluation of the feasibility of using synthetic data to compensate for missing input channels so that existing AI models could be clinically established. We hypothesized that synthetic data generated by GANs in combination with other original contrast images can support sufficiently well existing segmentation models for automatic multi-label segmentations of brain tumors. The validity and repeatability of the segmentation model when subjected to all original images with more or fewer inputs and when subjected to synthetic images shall be evaluated individually.

## 2 Materials and methods

### 2.1 Materials

For this study, the brain tumor segmentation (BraTS) 2021 dataset (15), publicly made available by the joint organization of the Radiological Society of North America, the American Society of Neuroradiology, and the Medical Image Computing and Computer Assisted Interventions society, was used for the training, validation, and testing of the algorithm. The BraTS2021 dataset comprised 1,251 patients with multi-institutional pre-operative baseline mpMRI scans, including 3D sequences such as T_1_w, T_2_w, T_1_CE, and FLAIR, all presented in Neuroimaging Informatics Technology Initiative (NIfTI) format. The multi-label segmentation consisting of edema (ED), necrotic tumor (NC), and contrast-enhanced tumor (CET) was provided for each patient and used as ground truth (GT) for automatic analysis. All the images were provided rigidly aligned, resampled to 1 mm^3^ isotropic resolution, and skull-stripped. Before training the models, the images were subjected to several pre-processing steps including the generation of a region of interest (ROI) mask and whole tumor (WT) mask; relabeling sub-regions of the GT mask to standardized voxel value as expected by automatic algorithm; co-registering all the input modalities, GT, and ROI mask to have same anatomical presentation with identical image size (240 × 240 × 155); and standardizing to zero mean and unit variance. The dataset was later randomly divided into subsets of 1,000 and 251 patients for training plus validation and testing of algorithms, respectively.

### 2.2 Methods

**Figure 1:**
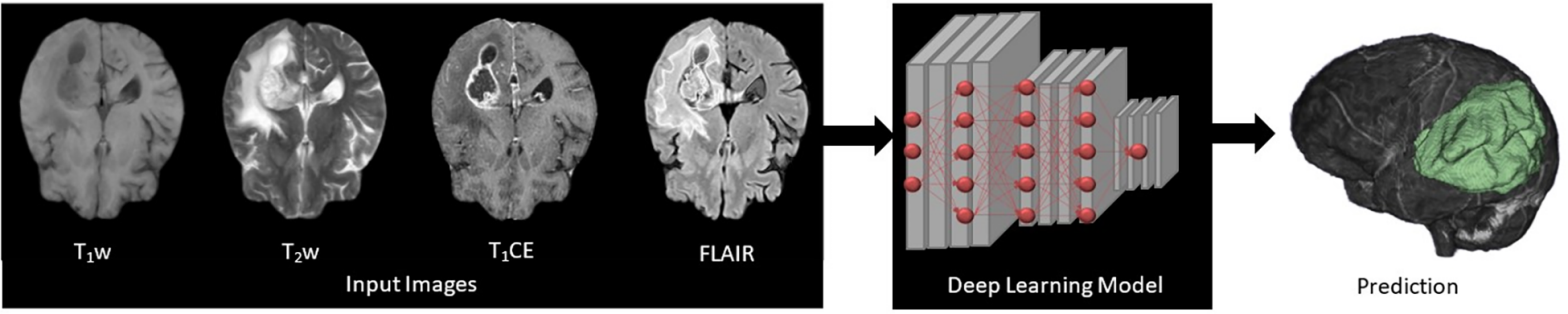
Four input (T_1_w, T_2_w, T_1_CE, and FLAIR) images subjected to deep learning (DL)-based 3D convolutional neural networks presenting feature extraction and classification for the automatic multi-label segmentation of brain tumors.

**Figure 2:**
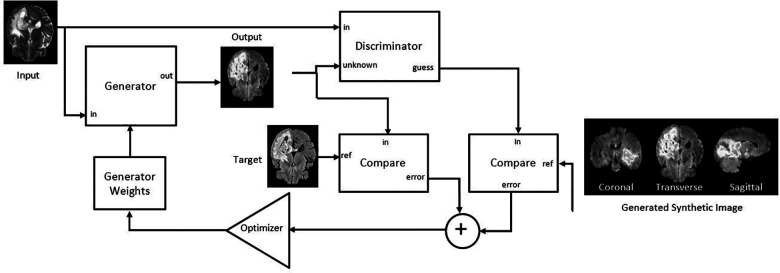
Adapted Pix2PixNIfTI architecture of conditional generative adversarial network (cGAN) for the creation of target image (FLAIR in the figure) using reference image (T_2_w in the figure) (20).

**Figure 3:**
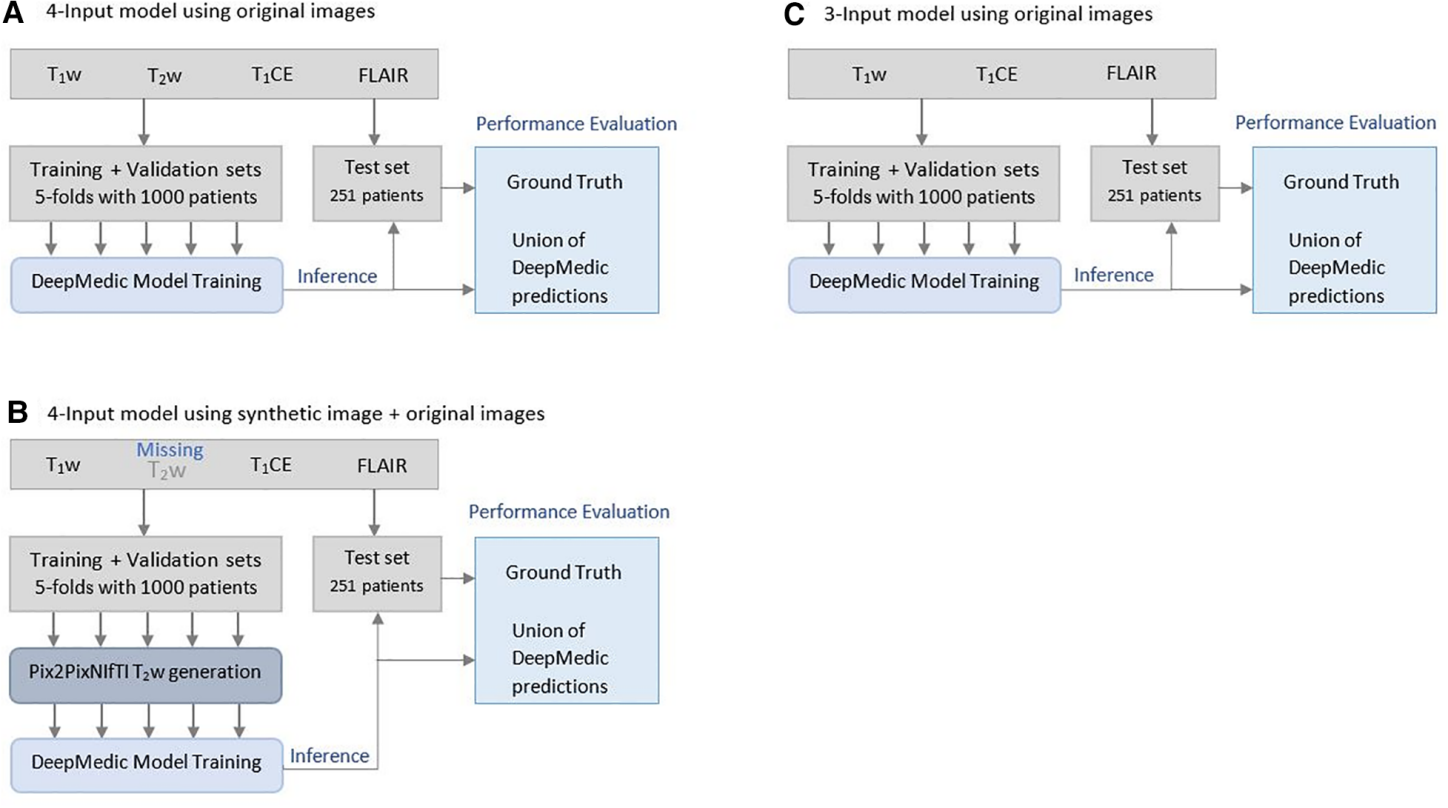
Illustration of Brain Tumor Segmentation (BraTS) 2021 data split and five-fold cross-validation of 1,251 patients for deriving multi-class automatic segmentation using the (**A**) four-input DeepMedic model using original images, (**B**) for generation of missing input using the 3D Pix2PixNIfTI model with inference to four-input DeepMedic model, and (**C**) for the three-input DeepMedic model using original images.

The mpMRI BraTS2021 dataset was subjected to two DL-based methods, primarily for multi-class automatic segmentation of brain lesions and secondary for image-to-image translation purposes as shown in Figure 1 and Figure 2. The DeepMedic algorithm, which is known for its configurable multi-resolution pathways to extract features and classify them, was employed as a benchmark for the multi-label segmentation (16, 17), whereas for image-to-image translation, the tool Pix2PixNIfTI (18, 19) was implemented. Pix2PixNIfTI is a 3D single-input single-output variant of conditional GAN architecture that learns the mapping between two MRI sequences to generate an approximated map of the target image. The 1,000-patient subset of the BraTS2021 dataset was further divided into sets of 800 and 200 patients, respectively, for training and validation. The split of training and validation data followed five-fold cross-validation, a process in which the intended dataset is randomly split by 80% and 20% into training and validation sets, respectively, for the specified number of folds (*n* = 5 for this study) as shown in Figure 3. All the CNN were first evaluated using five-fold cross-validation subsets and then were tested individually using a cohort of 251 patients. All the algorithms were executed on a Linux workstation equipped with a graphics processor unit to enable fast processing of the images.

### 2.3 Data processing

To serve the primary goal of the study, the segmentation model, DeepMedic, was trained by using four input images (T_1_w, T_2_w, T_1_CE, and FLAIR) with a batch size of 10 and 35 epochs (referenced as the four-input model). The same algorithm with identical batch size and epochs was later also trained with three input images of four combinations (referenced as the three-input model) as listed in Table 1.

**Table 1:** Deepmedic model trained with original input image combinations for multi-label automatic segmentations of brain tumors.

DeepMedic	
Four-input model	Three-input model
T_1_w, T_2_w, T_1_CE, FLAIR	T_1_w, T_2_w, T_1_CE
	T_1_w, T_2_w, FLAIR
	T_1_w, T_1_CE, FLAIR
	T_1_CE, T_2_w, FLAIR

To test the hypothesis of providing missing input image for a four-input segmentation model, the synthetic image-generating 3D model (Pix2PixNIfTI) was trained and tested using the same subset of training, validation, and test dataset in a five-fold cross-validation manner with a batch size of 1 and total 100 epochs. The intended image was generated using other available images (reference image) as specified in Table 2.

**Table 2:** The Pix2pixnifti model generated T_1_w, T_2_w, T_1_CE, and FLAIR images using other images.

Target image	Reference image		
T_1_w	T_1_CE	T_2_w	FLAIR
T_2_w	T_1_w	T_1_CE	FLAIR
T_1_CE	T_1_w	T_2_w	FLAIR
FLAIR	T_1_w	T_2_w	T_1_CE

Each intended target image (e.g., T_1_w) was generated using three available reference images (e.g., T_1_CE, T_2_w, FLAIR) giving rise to a total of 12 approximated images of all original input images.

The generated images were later used within a DeepMedic model by replacing one of the original images in a five-fold cross-validation manner as indicated in Table 3.

**Table 3:** The combination of generated T_1_w, T_2_w, T_1_CE, and FLAIR images with other original images provided to the four-input DeepMedic segmentation model.

DeepMedic			
Synthetic T_1_w	Synthetic T_2_w	Synthetic T_1_CE	Synthetic FLAIR
T_1_w_T1CEgen_,T_2_w,T_1_CE,FLAIR	T_1_w,T_2_w_T1gen_,T_1_CE,FLAIR	T_1_w,T_2_w,T_1_CE_T1gen_,FLAIR	T_1_w,T_2_w,T_1_CE,FLAIR_T1gen_
T_1_w_T2gen_,T_2_w,T_1_CE,FLAIR	T_1_w,T_2_w_T1CEgen_,T_1_CE,FLAIR	T_1_w,T_2_w,T_1_CE_T2gen_,FLAIR	T_1_w,T_2_w,T_1_CE,FLAIR_T2gen_
T_1_w_FLgen_,T_2_w,T_1_CE,FLAIR	T_1_w,T_2_w_FLgen_,T_1_CE,FLAIR	T_1_w,T_2_w,T_1_CE_FLgen_,FLAIR	T_1_w,T_2_w,T_1_CE,FLAIR_T1CEgen_

The subscripts denote the image on which the generated image was based, e.g., “FLAIR_T1gen_” denotes a FLAIR image that was generated from a T_1_w image.

### 2.4 Statistical analysis

The statistical analysis involved three steps, primarily determining the accuracy, precision, and sensitivity of segmentation models in evaluating multi-label predictions as close as possible to the GT. The similarities and reproducibility between GT and automated segmentation of five-fold models were assessed using the dice coefficient for both WT and individual tumor labels (NC, ED, CET). The dice were primarily evaluated for individual patients for all classes considering the GT segmentation and unionized using five predictions from cross-validation folds and later averaged over all patients.

Following dice coefficient measurement, differences between tumor sub-regions were tested for statistical significance using a standard paired *t*-test. The comparison included automatically generated segmentations of two models, one using a combination of all original images and one model substituting an original image with a synthetically generated one. Statistical significance was set to the value *p* < 0.05 for unit segmentation and then was adjusted for multiple comparisons (Bonferroni correction).

Finally, mean squared error (MSE), a quantitative measure of image quality, was evaluated for all synthetic images to assess the average error reflecting the difference between the original and predicted images for the brain volume of the generated image.

## 3 Results

As a part of the statistical assessment, the primary analysis was carried out to determine the best-performing model in achieving multi-class segmentation among four-input trained DL-based algorithms as the best-case scenario when all the original images are available. For model evaluation, common measures such as dice coefficient per class and for WT, with accuracy, sensitivity, and specificity were calculated.

### 3.1 Prediction evaluated using a four-input DeepMedic model using an original image

In the standard evaluation of ensembled five-fold DeepMedic model prediction generated using all original images, the dice coefficient observed for individual classes was high for ED and CET (0.86 ± 0.13 and 0.86 ± 0.20, respectively), but moderately acceptable dice were measured for NC with 0.76 ± 0.29 score. The assessment of WT provided the best dice score for prediction derived using a four-input model with a value of 0.93 ± 0.06. The model predicted the segmentation with accuracy, precision, sensitivity, and MSE for all classes as interpreted in Table 4.

**Table 4:** Mean dice scores with SD, accuracy, precision, sensitivity, and MSE evaluated for individual tumor sub-regions and whole tumor for predictions evaluated using a four-input DeepMedic model with original input images.

Parameter	Dice score			
	Necrotic tumor core	Edema	Contrast-enhanced tumor	Whole tumor
Dice coefficient ± SD	0.76 ± 0.29	0.86 ± 0.13	0.86 ± 0.20	0.93 ± 0.06
MSE	0.01	0.01	0.01	0.001
Accuracy	0.99	0.99	0.99	0.99
Sensitivity	0.87	0.87	0.86	0.96
Specificity	0.99	0.99	0.99	0.99

### 3.2 DeepMedic prediction evaluated using synthetic image inference

In the evaluation of five-fold ensembled DeepMedic-derived predictions for each class, the predictions using synthetic T_1_CE images were observed to have the lowest dice scores per class, especially for NC and CET, when compared with other predictions derived using inference of other synthetic images. The predictions generated using the synthetic T_1_CE inference (either using T_1_w, T_2_w, or FLAIR contrast as reference for synthetic T_1_CE generation) all had a similar range of dice score per class (range of dice NC, 0.27–0.31; ED, 0.62–0.64; CET, 0.12–0.23; WT, 0.82–0.83). Comparatively, synthetic FLAIR and synthetic T_1_w DeepMedic predictions were observed to have moderately acceptable dice scores per class. For synthetic FLAIR DeepMedic prediction, the highest dice scores per class were achieved when FLAIR images were generated using T_2_w images (NC, 0.70 ± 0.31; ED, 0.56 ± 0.21; CET, 0.82 ± 0.22). On the other hand, for synthetic T_1_w DeepMedic prediction, the dice scores per class were observed to have a similar range when compared with predictions generated using synthetic T_1_CE, T_2_w, and FLAIR images, and very little differences were perceived between acquired predictions (NC, 0.64 ± 0.30; ED, 0.77 ± 0.18; CET, 0.79 ± 0.23). The best prediction using the DeepMedic model with synthetic images was observed with synthetic T_2_w images, yielding predictions with dice scores similar to predictions based on original images. The DeepMedic predictions using synthetic T_2_w images generated from a reference image of FLAIR, T_1_CE, or T_1_w led to dice scores per class in similar ranges and yielded the highest values when compared with other DeepMedic predictions using synthetic data (NC, 0.74 ± 0.30; ED, 0.81 ± 0.15; CET, 0.84 ± 0.21).

**Figure 4:**
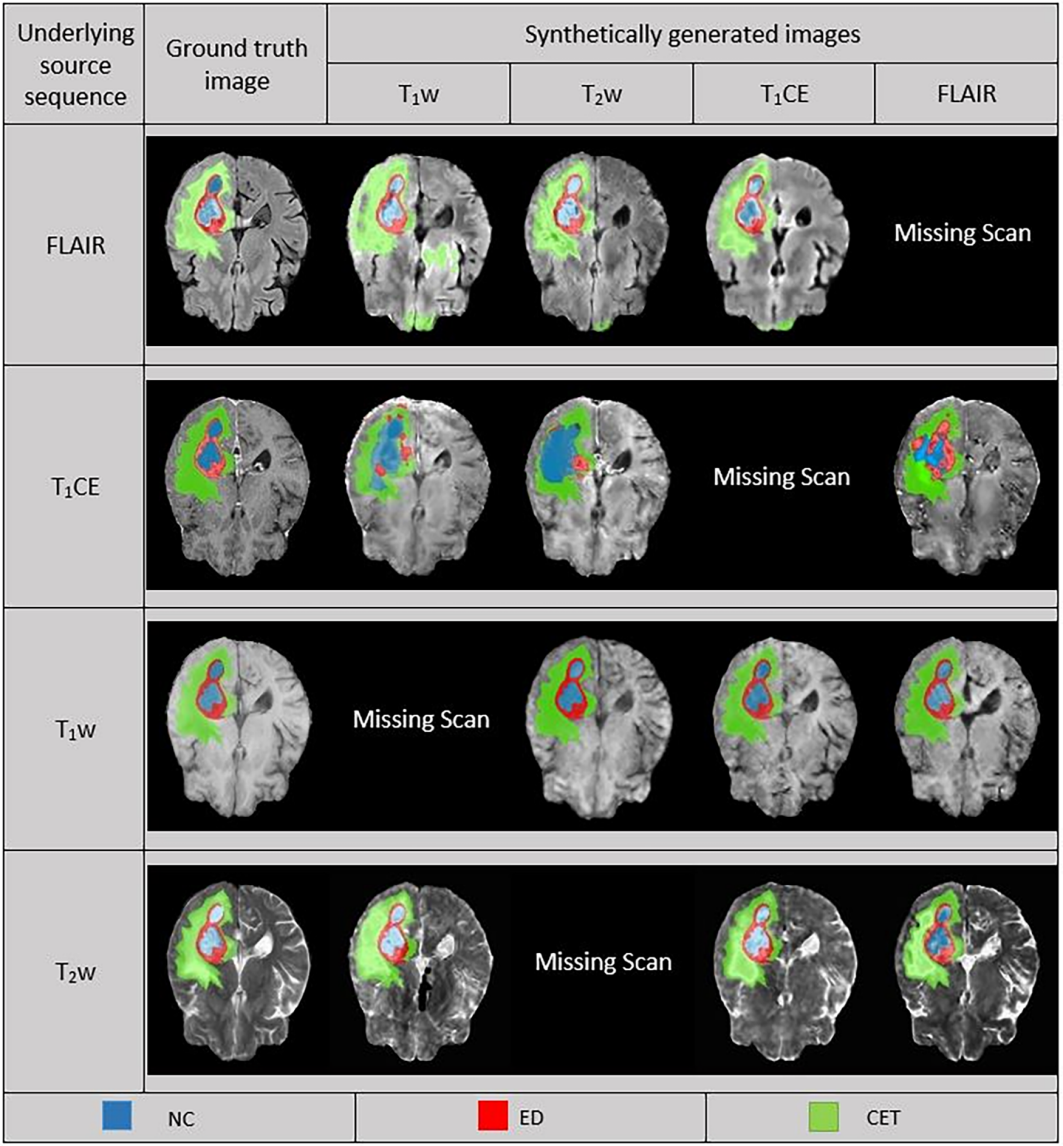
Comparison between ground truth (GT) and DeepMedic predicted multi-class segmentations derived from generated synthetic images using multiple contrasts, superimposed on their respective original and generated synthetic images. Grayscale for normalized generated synthetic images T_1_w, −3.0 to 9.0; T_2_w, −1.8 to 6.3; T_1_CE, −2.5 to 8.5; FLAIR, −2.0 to 6.5.

In the predictions of the five-fold ensembled DeepMedic for the WT assessment, some unexpected differences from regional evaluations were noticed. Using synthetic T_2_w image, DeepMedic predictions had the highest dice scores just like in the individual label evaluation (WT, 0.90 ± 0.08). The synthetic T_1_w DeepMedic prediction had the second-highest dice score of WT (WT, 0.85 ± 0.12); however, surprisingly the lowest dice score for WT was observed in synthetic FLAIR DeepMedic prediction (WT, 0.64 ± 0.19), while synthetic T_1_CE prediction had a comparatively higher dice score for WT evaluation (WT, 0.83 ± 0.13). The dice scores can be reviewed in Table 5, and for the visual representation of the segmentations, Figure 4 can be referred to.

**Table 5:** Mean dice scores with SD evaluated for individual tumor sub-region and whole tumor for predictions evaluated using applied inference of synthetic images generated with image-to-image translation method to DeepMedic model with combination of three original input images and one synthetic image, along with four-input DeepMedic prediction derived using original images for comparison purpose.

Target image	Reference image	Dice ± SD			
		Necrotic tumor core	Edema	Contrast-enhanced tumor	Whole tumor
FLAIR	T_1_CE	0.70 ± 0.32	0.49 ± 0.22	0.80 ± 0.23	0.64 ± 0.19
	T_1_w	0.69 ± 0.32	0.50 ± 0.21	0.80 ± 0.23	0.66 ± 0.17
	T_2_w	0.70 ± 0.31	0.56 ± 0.21	0.82 ± 0.22	0.70 ± 0.17
T_1_CE	FLAIR	0.27 ± 0.22	0.62 ± 0.17	0.23 ± 0.18	0.83 ± 0.13
	T_1_w	0.31 ± 0.25	0.64 ± 0.16	0.12 ± 0.11	0.83 ± 0.12
	T_2_w	0.29 ± 0.25	0.64 ± 0.16	0.21 ± 0.17	0.82 ± 0.13
T_1_w	FLAIR	0.63 ± 0.30	0.76 ± 0.18	0.79 ± 0.23	0.85 ± 0.12
	T_1_CE	0.66 ± 0.30	0.77 ± 0.18	0.80 ± 0.23	0.85 ± 0.12
	T_2_w	0.64 ± 0.30	0.77 ± 0.18	0.79 ± 0.23	0.86 ± 0.12
T_2_w	FLAIR	0.73 ± 0.30	0.82 ± 0.14	0.84 ± 0.21	0.90 ± 0.08
	T_1_CE	0.74 ± 0.30	0.81 ± 0.15	0.84 ± 0.21	0.90 ± 0.08
	T_1_w	0.74 ± 0.30	0.81 ± 0.15	0.84 ± 0.21	0.90 ± 0.09
Four-input original image DeepMedic prediction		0.76 ± 0.29	0.86 ± 0.13	0.86 ± 0.20	0.93 ± 0.06

All the dice scores are statistically significant for all tumor sub-regions (*p *< 0.00083).

### 3.3 Prediction evaluated using a three-input DeepMedic model using an original image

**Figure 5:**
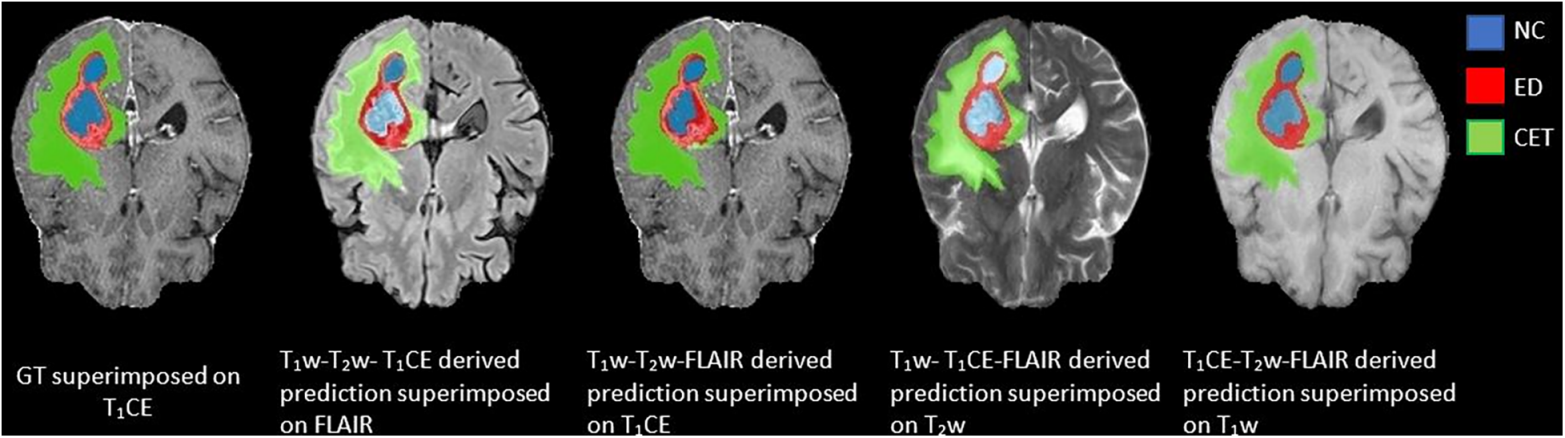
Predictions generated using a three-input trained DeepMedic model with original image combinations of T_1_CE–T_1_w–T_2_w, T_1_w–T_2_w–FLAIR, T_1_CE–T_1_w–FLAIR, and T_1_CE–T_2_w–FLAIR superimposed on missing input, presented with GT superimposed on T_1_CE image for comparison.

Among the four combinations of three-input trained DeepMedic model used for deriving multi-class brain tumor segmentation, the T_1_CE–T_2_w–FLAIR image combination had the highest dice scores for both individual classes as well as for WT assessment (NC, 0.77 ± 0.28; ED, 0.86 ± 0.12; CET, 0.85 ± 0.20; WT, 0.93 ± 0.06). On the other hand, predictions using the T_1_w–T_2_w–T_1_CE combination were observed to have slightly lower dice scores in comparison to the T_1_CE–T_2_w–FLAIR combination, but still the second best dice score for both WT and tumor sub-region evaluation using three-input model (NC, 0.76 ± 0.29; ED, 0.81 ± 0.14; CET, 0.85 ± 0.21; WT, 0.90 ± 0.07). One of the average quality dice scores was acquired with the T_1_w–T_2_w–FLAIR image combination; however, on the contrary, the combination was surprisingly efficient for WT assessment with dice scores in a similar range to T_1_CE–T_2_w–FLAIR combination (NC, 0.57 ± 0.29; ED, 0.79 ± 0.15; CET, 0.62 ± 0.23; WT, 0.92 ± 0.07). Another pair with average dice scores for WT and individual tumor class regions, especially for NC and CET was the T_1_w–T_1_CE–FLAIR image combination (NC, 0.61 ± 0.24; ED, 0.70 ± 0.12; CET, 0.70 ± 0.19; WT, 0.77 ± 0.07). For visual representation and numerical interpretation, Figure 5 and Table 6 can be referred to, respectively.

**Table 6:** Mean dice scores with SD evaluated for individual tumor sub-regions and whole tumor for predictions evaluated using a three-input DeepMedic model with multiple contrast combination of three original images and four-input DeepMedic prediction derived using original images for comparison purposes.

Three-input contrast combination for DeepMedic prediction	Dice ± SD			
	Necrotic tumor core	Edema	Contrast-enhanced tumor	Whole tumor
T_1_w–T_1_CE–FLAIR	0.61 ± 0.24	0.70 ± 0.12	0.70 ± 0.19	0.77 ± 0.07
T_1_w–T_1_CE–T_2_w	0.76 ± 0.29*	0.81 ± 0.14*	0.85 ± 0.21*	0.90 ± 0.07*
T_1_CE–T_2_w–FLAIR	0.77 ± 0.28*	0.86 ± 0.12*	0.85 ± 0.20*	0.93 ± 0.06*
T_1_w–T_2_w–FLAIR	0.57 ± 0.29	0.79 ± 0.15	0.62 ± 0.23	0.92 ± 0.07
Four-input original image DeepMedic prediction	0.76 ± 0.29	0.86 ± 0.13	0.86 ± 0.20	0.93 ± 0.06

The dice scores statistically not significant for tumor sub-regions are marked with a “*” sign (*p* > 0.0025).

### 3.4 Comparison of three-input predictions using original images and synthetic image predictions

To address the issue of missing input for four-input trained models, two approaches were implemented to create the prediction using DeepMedic. The primary method involving the generation of synthetic images in a four-input DeepMedic model could provide comparable results for different tumor sub-regions and slightly improved results for WT. The usage of synthetic T_1_w and T_2_w images in the four-input DeepMedic model was observed to predict tumor sub-regions of ED and CET proportionately but notably underpredicted the NC regions. On the other hand, predictions based on synthetic FLAIR images were averagely acceptable for CET and NC but perceived to be undermining the ED sub-region. The T_1_CE predictions were significantly under-evaluated for the NC and CET regions and had average dice scores for the ED region. On the contrary, predictions of the WT volume yielded high dice scores for all synthetically generated images except for FLAIR.

Similar to synthetic images, predictions generated using the second approach of the three-input trained DeepMedic model provided similar results to some extent. The predictions with the highest dice scores were observed for T_1_w–T_2_w–T_1_CE and T_1_CE–T_2_w–FLAIR image combinations, yielding predictions as close as to the predictions based on the four-input DeepMedic model of original images, for both individual class and WT. On the other hand, the T_1_w–T_1_CE–FLAIR and T_1_w–T_2_w–FLAIR combinations led to average prediction per class but were efficient for WT segmentation. The dice scores evaluated for DeepMedic predictions using synthetic images in the four-input model were statistically significantly different for all regions (*p* << 0.00083). On the other hand, DeepMedic predictions of the three-input model for the T_1_CE–T_2_w–FLAIR and T_1_w–T_1_CE–FLAIR combinations were found to be not statistically significant (*p* > 0.0025) while T_1_w–T_2_w–T_1_CE and T_1_w–T_2_w–FLAIR combinations were observed to be significant for all tumor sub-region (*p* << 0.0025).

### 3.5 Quantitative analysis of synthetic images and correlation with volumetric prediction

To measure the level of accuracy attained by the generated synthetic images when compared to their corresponding GT, we assessed the (MSE) for each synthetic image in a five-fold cross-validation manner and subsequently averaged it to determine the overall mean across all subsets. The MSE evaluated for each target image using different reference images was observed to have comparable extent of values. A higher MSE suggests a larger disparity between the synthetic image and GT. In comparison, the T_1_CE synthetic images were noticed to have the highest MSE relative to other synthetic images, while T_1_w synthetic images had a slightly lower but second-highest MSE. The synthetic images of FLAIR and T_2_w comparatively have lower MSE.

To analyze the impact of synthetic images in deriving multi-class prediction using the four-input segmentation model, we evaluated the Pearson and Spearman correlation between the MSE of synthetic image and the dice coefficient of its respective four-input model derived prediction for each class as well as for WT region. Both methods unanimously indicated the negative correlation between synthetic images and their respective predictions suggesting an inverse relationship between these two variables as expected. The inverse relationship implies that, as the MSE of synthetic images increases, the dice coefficient of tumors tends to decrease, suggesting higher errors in the synthetic images are associated with lower dice, which implies poorer segmentation accuracy. For numerical interpretation of MSE of synthetic images and correlation, Table 7 can be referred to.

**Table 7:** Mean MSE of individual synthetic image calculated for multiple reference images along with evaluated corresponding Pearson and Spearman correlation coefficient of synthetic image MSE and dice score of respective four-input model prediction for multi-class and whole tumor sub-region.

Target image	Reference image	Synthetic image MSE	Pearson coefficient		Spearman coefficient	
			Multi-class	Whole tumor	Multi-class	Whole tumor
FLAIR	T_1_CE	0.517307	−0.09747	−0.07988*	−0.17191	−0.22446
	T_1_w	0.494333	−0.10610	−0.08877*	−0.15752	−0.15511
	T_2_w	0.342234	−0.07842	−0.00643*	−0.15566	−0.20277
T_1_CE	FLAIR	0.896027	−0.01530*	0.09458*	−0.03816*	0.04452*
	T_1_w	1.052703	0.01256*	0.13154	0.00432*	0.11043*
	T_2_w	0.972855	−0.00596*	0.11747*	−0.02017*	0.12646
T_1_w	FLAIR	0.764378	−0.05202*	−0.08146*	−0.11485	0.00669*
	T_1_CE	0.628514	−0.02677*	0.02138*	−0.02819*	0.09077*
	T_2_w	0.845746	−0.05245*	−0.07035*	−0.13704	−0.05480*
T_2_w	FLAIR	0.454605	−0.08700	−0.02092*	−0.11868	−0.16521
	T_1_CE	0.383218	−0.11272	−0.04269*	−0.17125	−0.20306
	T_1_w	0.439892	−0.12217	−0.05132*	−0.15581*	−0.18053

The correlation coefficient statistically not significant for tumor sub-regions is marked with a “*” sign (*p* > 0.05).

## 4 Discussion

The study mainly focuses on mitigating issues of missing input channels for automatic segmentation, for which two approaches were adopted. The primary approach included the generation of missing input channels for automatic segmentation using a variant of GAN architecture. Another approach investigated the existing multi-class segmentation model trained with fewer input channels, in this case, three input channels.

In the primary approach, we explored the efficiency of synthetic images generated using the 3D Pix2PixNIfTI algorithm in a single-input, single-output manner for all available reference images to determine the best surrogate image for the segmentation model when certain input is missing. We assessed the outcomes based on a visual and a quantitative evaluation. In a visual qualitative assessment of synthetic images, the generated images were observed to be influenced by the type of input reference image chosen for synthesis but perceived as nearly indistinguishable. For example, images generated using FLAIR and T_2_w were slightly hypointense while images generated by T_1_w and T_1_CE were hyperintense. In a quantitative assessment, the best image-to-image translation was observed for synthetic T_2_w image (T_2_w_T1Cegen_ dice: NC, 0.74 ± 0.30; ED, 0.81 ± 0.15; CET, 0.84 ± 0.21; WT, 0.90 ± 0.08), following T_1_w and FLAIR, though synthetic T_1_CE image could not be accepted as a replacement since the dice for NC and CET are most undermined compared to other synthetic image used evaluated predictions. Similar observations for synthetic T_1_CE for single-input single-output architecture were reported by Sharma and Hamarneh (21), Lee et al. (22), and Li et al. (23) who used other variants of the GAN-based algorithm. In our quantitative evaluation of synthetic images, the findings suggest a higher error for synthetic T_1_CE images and slightly less but still comparably high MSE for synthetic T_1_w images implying unsatisfactory synthesis of generated images. On the other hand, synthetic FLAIR and T_2_w images indicated lower MSE values, suggesting higher accuracy in translating original image properties to synthetic images.

In our quantitative assessment, we observed that the choice of reference image when generating synthetic images for multi-class prediction delivered ambiguous results, meaning that it is unclear which reference image should be used and it is contingent on the specific situation at hand. For T_1_CE, the prediction using synthetic T_1_CE yielded decent dice for the ED region when T_1_w and T_2_w were used as reference images but witnessed severely undermined dice for CET using T_1_w as a reference image compared to other images, while the lowest dice for NC as observed for FLAIR when used as reference. Therefore, based on the application, the synthetic T_1_CE images can be implemented for segmentation. For T_2_w, the highest dice scores per class and for WT were found for DeepMedic predictions using surrogate based on either T_1_w, T_1_CE or FLAIR. These findings suggest that GAN successfully translated the physical properties to the target image, aiding multi-class automatic segmentation of tumor with decent dice (NC, 0.74 ± 0.30; ED, 0.81 ± 0.15; CET, 0.84 ± 0.21; WT, 0.90 ± 0.09). Whereas for T_1_w, the evaluated predictions were observed to have a slight declination in the dice scores per class and for WT compared to synthetic T_2_w, especially for the NC region (reduction of dice NC, 10%, ED, 4%; CET, 5%; WT, 5%). Similarly, DeepMedic predictions using synthetic FLAIR images had resembling dice scores except for ED and WT, which were the lowest values observed (NC, 0.70 ± 0.32; ED, 0.49 ± 0.22; CET, 0.80 ± 0.23; WT, 0.64 ± 0.19). Both DeepMedic predictions using synthetic T_1_w and FLAIR images were observed to have relatively low dice scores for the NC and ED, respectively, interpreting that using the reference contrast image-to-image method could not entirely translate the tissue properties to the magnitude of the target image. On the other hand, the predictions making use of synthetic T_1_CE were observed most compromising results with the lowest dice scores for NC and CET while producing decent dice scores for ED and WT. The image-to-image translation could fairly synthesize non-enhancing T_2_–FLAIR hyperintense regions of the tumor; however, contrast-enhancing and necrotic portions of the tumor were not translated to a target image. In clinical practice, the administration of contrast agents enhances the interstitial and intravascular properties of the tissue, which theoretically can not be artificially synthesized or produced by non-contrast imaging sequences. To assess the impact of synthetic images on segmentation ability, we conducted a correlation analysis that highlighted the inverse relationship between the MSE of synthetic images and the dice score of segmentation model prediction. In our analysis, the highest MSE was observed for synthetic T_1_CE, and the lowest dice scores were also evaluated for synthetic T_1_CE-derived prediction, implying the negative correlation that indicates the inverse relationship between these two variables, suggesting that the presence of a higher degree of measured error in the synthetic image reflects poor quality, which greatly affects the segmentation accuracy and precision and vice versa. The assumption is consistent for other synthetic images as well, which are observed to have lower MSE but higher dice for evaluated predictions. Depending on the degree of error present in the synthetic image proportional segmentation efficiency would be impacted.

On the contrary, a study conducted by Jayachandran Preetha et al. (24) suggested that it is possible to generate fair quality post-contrast images by using single-input and/or multi-input contrast images and cGAN-based architecture, a variant inspired by pix2pix (12). Their study explored two architectures and concluded that the cGAN-based algorithm was superior to the other while including diffusion-weighted imaging for better results was not found to have any significance. In the study of Li et al. (23), the feasibility of generating a post-contrast T_1_ sequence was tested with two architectures for a single and multi-input model, out of which the multi-input trained algorithm performed most well. In regards to the multi-input model, it is always not feasible for all input images to be available for the synthesis of the target image, yet for special cases like T_1_CE, the method can be adapted using a multi-input GAN-based model. A comparative study done by Conte et al. (25) showed the feasibility of a 2D pix2pix model, for generating synthetic images of FLAIR and T_1_w images and acquired quite efficient results for ED and CET region of tumor automatic segmentation. The study only focuses on generating FLAIR and T_1_w images from T_2_w and T_1_CE images, respectively, and does not account for generating other images using different types of contrasts. Additionally, the efficiency of synthetic images in deriving automatic segmentation was only tested for ED and CET sub-regions but the NC and WT portions were left out. Thomas et al. (26). tested 2D many-to-many mapping approach using a nnUNet variant as architecture for the synthesis of target images. This approach was not yet adapted before which includes multiple combinations of reference images and masks with copies of images in variation and therefore paved a new way for multi-class segmentation. However, the study was implemented on a relatively small sample (*n* = 231; e.g. using synthetic T_2_ ED, 0.74; CET, 0.80; WT, 0.90) excluding assessment of the NC region and was limited to generating 2D images. Another study by Zhou et al. (27), tested a U-Net variant as architecture to generate synthetic images. The study tested two methods of segmentation where replacing missing input using synthetic images was used as a conventional method and another method involved correction for a segmentation model that uses available images by adding components (multi-source correlation, conditional generator, and generator without condition constraint). The resultant outcome was measured for tumor core (TC), CET, and WT and was observed to be relatively low (e.g., dice of predictions using synthetic FLAIR_T2gen_ TC, 0.54; CET, 0.68; WT, 0.55) compared to our study (e.g., dice of predictions using synthetic FLAIR_T2gen_ NC, 0.70 ± 0.31; ED, 0.56 ± 0.21; CET, 0.82 ± 0.22; WT, 0.70 ± 0.17) when missing input channels were replaced with a synthetic image but were observed to have increased dice scores when tested with a combination of other components (e.g., dice of predictions using synthetic FLAIR_T2gen_ TC, 0.85; CET, 0.77; WT, 0.84).

In the following stage of the study, we investigated a secondary approach concerning missing input issues. For this purpose, we trained a DeepMedic model with three inputs, in which T_1_w–T_1_CE–T_2_w and T_1_CE–T_2_w–FLAIR contrast image combinations produced similar results to the predictions generated by the four-input DeepMedic model trained with original images. We noticed that both combinations had T_1_CE and T_2_w as common input channels, thus indicating that these are essential sequences to derive accurate multi-class segmentation. The conclusion is further supported by the observations of reduced dice scores per class for predictions based on the T_1_w–T_1_CE–FLAIR and T_1_w–T_2_w–FLAIR image sets, as the model was trained with an image set carrying either only T_1_CE or T_2_w alone, combining T_1_w and T_2_w derivative (FLAIR). The pair T_1_w–FLAIR seems inefficient together in the presence of either T_1_CE or T_2_w to draw comparable results for automatic segmentation. DeepMedic model trained with T_1_w–T_1_CE pair and any other contrast was observed to have a fairly decent estimation for NC core, again suggesting correlation to the selection of images (T_1_w–T_1_CE) for the training. Fundamentally, T_1_CE is known for identifying intrinsic perfusion characteristics of tissue like permeability indicating active tumor with healthy vascular supply, while T_2_w–FLAIR enhances subcutaneous fat and water-based tissue on the image, reflecting vasogenic or infiltrative nature of the tumor (edema), and T_1_w is most sensitive in the detection of damaged adipose tissue (1). Depending on the choice of combination of images selected for training of the model, a strong influence was observed on the generated prediction of the respective model reflecting tumor properties based on characteristics of the selected sequence of imaging. For example, DeepMedic prediction generated by T_1_w–T_2_w–FLAIR yielded a higher estimate for ED but a reduced dice value for other sub-regions comparatively, reflecting that T_2_w and FLAIR are important for ED estimation, but not for other sub-regions. We did not find any study with similar theory and observation for the DeepMedic algorithm but rather for other segmentation algorithms (28).

In our study, we found excellent multi-class segmentation results using three-input DeepMedic models when one of the four input sequences was missing especially for missing T_1_w or FLAIR images. However, if only a four-input segmentation model is available, synthetic images can also be used to replace a single-input channel for the prediction of multi-class segmentation or the WT area, although with slightly underestimated predictions. The concurrent paragraph summarizes the recommendations based on our findings. When the T_1_CE sequence is not available, it is best to use a three-input model (T_1_w–T_2_w–FLAIR), which fairly evaluates tumor sub-regions but yields a high dice score for the WT region compared to that with the four-input model (NC, 0.57 ± 0.29; ED, 0.79 ± 0.15; CET, 0.62 ± 0.23; WT, 0.92 ± 0.07). However, if only a four-input model is available, either T_2_w or FLAIR can be used to generate a synthetic T_1_CE image, especially for the segmentation of the ED region. In case of a missing T_2_w sequence, we recommend using the four-input segmentation model with a synthetic image generated using either T_1_CE, T_1_w, or FLAIR, as all of them provided equally high dice scores for different tumor sub-regions and WT as well (NC, 0.74 ± 0.30; ED, 0.81 ± 0.15; CET, 0.84 ± 0.21; WT, 0.90 ± 0.09). For a missing FLAIR sequence, a three-input segmentation model (T_1_w–T_1_CE–T_2_w) performed better in comparison to a four-input segmentation model for all classes (NC, 0.76 ± 0.29; ED, 0.81 ± 0.14; CET, 0.85 ± 0.21; WT, 0.90 ± 0.07). However, in the absence of a three-input model, the T_2_w image shall be used as a reference for generating a synthetic FLAIR image for multi-class segmentation. When T_1_w is missing, we assessed that a three-input segmentation model (T_1_CE–T_2w_–FLAIR) showed promising results per class and for WT with dice NC, 0.76 ± 0.29; ED, 0.81 ± 0.14; CET, 0.85 ± 0.21; and WT, 0.90 ± 0.07. If a three-input segmentation model is not available, we recommend using the T_1_CE image to generate a synthetic T_1_w image for a four-input multi-class segmentation model.

Albeit promising results, there are a few limitations to our study. We did not test any alternative single-input synthetic image-generating algorithm to obtain 3D surrogate images and rather investigated the relatively new variant of the pix2pix algorithm with promising results to the best of our knowledge. To identify the true conceivable potential of the Pix2PixNIfTI model in generating synthetic images further evaluation and optimization will be needed. Additionally, we abstained from testing multi-input synthetic image-generating algorithms due to the restricted feasibility of all input images being available in the clinical establishment. Moreover, we did not examine other CNN for less input channels application or using images other than the four typical contrasts and as such limits its applicability to this purpose. Furthermore, we did not evaluate the model’s efficiency to recognize and account for the absence of one of the tumor sub-regions if were missing. Despite these limitations, our study investigated the best configuration for multi-class segmentation using original images for conventional and moderately new models. As we expected, the DeepMedic model performed quite well in predicting multi-class segmentation outperforming the manual method in saving time while delivering comparable segmentation performance, and the obtained results were comparable with other studies using the same segmentation model (16, 29–31). Additionally, we successfully examined which image combinations complement each other to aid the segmentation of different tumor sub-regions with an incomplete set of input channels. Further, our study explored which input contrast generated the best replica of the target image by training the model multiple times with various individual input images to acquire a target synthetic image and investigated its impact on tumor sub-regions during automatic segmentation.

In summary, we discovered that it is feasible to use a DL-based model for multi-class segmentation as well as for the generation of synthetic images. Depending on the choice of available image and method, fairly accurate segmentation can be achieved either for WT, per class, or for both using original images. However, with lesser input or using a synthetic image for the DeepMedic model, a slightly reduced dice score per class for evaluated predictions would be achieved with an exception for synthetic T_1_CE prediction which witnessed the most undermined evaluation for individual tumor regions. Although for global assessment of diseased regions using WT volume, both methods can be employed with more accuracy and precision. In the future, we plan to compute the validity of the DeepMedic model using a high-quality external patient cohort, to discover the reasonable performance of the model in achieving multi-class segmentation in the next step of the study as secondary testing before clinical implementation. Also, new segmentation models such as nnUNet can be also tested as comparable segmentation models. Furthermore, we would try to optimize the existing GAN-based model in generating missing input for the segmentation model to enhance the results and for the method to be considered for implementation in the clinical routine. Additionally, the efficiency of a multi-input approach for generating comparable T_1_CE images can also be explored.

## Data Availability

Publicly available datasets were analyzed in this study. This data can be found here: https://www.kaggle.com/datasets/dschettler8845/brats-2021-task1/data SynapseID: syn25829067.
